# Amelioration of the hepatotoxic effects of nonsteroidal drugs using vitamin C and determination of their relationship with the lipid profile

**DOI:** 10.1016/j.jtumed.2021.11.003

**Published:** 2021-12-13

**Authors:** Manal N. Al-Hayder, Tamadir H.W. Aledani, Rawaa S. Al-Mayyahi

**Affiliations:** a Department of Pharmacology and Toxicology, College of Pharmacy, University of Basrah, Basrah, Iraq; b Department of Clinical Laboratory Sciences, College of Pharmacy, University of Basrah, Basrah, Iraq

**Keywords:** تسمم الكبد, هيستوباثولوجيا الكبد, الشاكلة الشحمية, الأدوية غير الستيرويدية المضادة للالتهاب, فيتامين سي., Hepatotoxicity, Histopathology, Lipid profile, Nonsteroidal anti-inflammatory drugs, Vitamin C

## Abstract

**Objective:**

Despite the various clinical benefits of nonsteroidal anti-inflammatory drugs, their frequent and prolonged use has led to numerous health risks, including hepatotoxicity. Hepatotoxicity mediated by oxidative stress can affect the lipid profile. The objective was to investigate whether post-treatment with vitamin C can ameliorate the effects of diclofenac and naproxen in the livers of prepubertal rats and to highlight their relationship with lipid profile.

**Methods:**

Forty prepubertal female albino rats were distributed among the control group, the diclofenac-administered group (5 mg/kg/day), and the naproxen-administered group (50 mg/kg/day). This study included two phases. In Phase 1, only five rats from each group were dissected after 21 days of oral administration to assess the hepatotoxic effects of nonsteroidal drugs. In Phase 2, five of the remaining animals in each intervention group were post-treated with 25 mg/kg/day of vitamin C for an additional 21 days. After the administration and post-treatment, serum biochemical parameters and histopathological signs were evaluated.

**Results:**

Extreme elevation in the levels of aspartate and alanine aminotransferases was observed in the diclofenac and naproxen groups compared with those in the control (*p* < 0.001). In addition, the levels of high- and low-density lipoproteins were significantly impacted in these drug groups (*p* < 0.01, *p* < 0.05 respectively). Several pathological signs in the liver histology were observed in both drug groups. After post-treatment with vitamin C, noticeable amelioration of these alterations was observed. There were slightly elevation in the liver enzymes and insignificant increase and decrease in the high and low-density lipoproteins respectively.

**Conclusion:**

Vitamin C post-treatment ameliorated the hepatotoxicity induced by diclofenac sodium and naproxen.

## الملخص

**أهداف البحث:**

الاستعمالات المتكررة والطويلة الأمد للأدوية غيرالستيرويدية المضادة للالتهاب يؤدي الى عدة مخاطر، على الرغم من منافعها السريرية. تسمم الكبد هو أحد تلك المخاطر الذي يتسبب بواسطة الإجهاد التأكسدي ويمكن ان يؤثر على الشاكلة الشحمية. الهدف من الدراسة هو التحقق من إمكانية استعمال فيتامين سي كمضاد تأكسد قوي في عكس التأثيرات الضارة للديكلوفيناك صوديوم والنابروكسين في أكباد الجرذان غير البالغة وتسليط الضوء على علاقة تلك التاثيرات بالشاكلة الشحمية.

**طرق البحث:**

استخدمت الدراسة 40 أنثى جرذ غير بالغة. قسمت إلى 3 مجموعات: المجموعة الأولى تم إعطاؤها يوميا عن طريق الفم خمسة ملغرام لكل كيلوغرام من وزن الجسم ديكلوفيناك صوديم (15 جرذ)، والمجموعة الثانية تم إعطاؤها 50 مليغرام لكل كيلوغرام نابروكسين (15 جرذ) لمدة 21 يوم، أما المجموعة الثالثة المكونة من 10 جرذان فاستخدمت للمقارنة كمجموعة شاهدة. بعد نهاية الأسبوع الثالث للتجريع، تم معالجة خمسة جرذان من مجموعة الديكلوفيناك وخمسة جرذان من مجموعة النابروكسين بفيتامين سي (25 مليغرام/ كيلوغرام) يوميا عن طريق الفم. بعد ذلك، قدرت المعايير الكيميائية الحيوية لمصل الدم باستعمال عدد تجارية واستعمل إجراء روتيني للبحث النسيجي المرضي.

**النتائج:**

تم تسجيل ارتفاع شديد في مستويات ناقلة أمين الاسبارتات وناقلة أمين الالانين في مجموعتي الديكلوفيناك والنابروكسين بالنسبة إلى مجموعة المقارنة. كذلك، تأثرت البروتينات الشحمية العالية والمنخفضة الكثافة معنويا في المجموعتين الدوائية. أما بالنسبة لأنسجة الكبد فقد لوحظت علامات مرضية عديدة في كل من المجموعتين الدوائية. بعد المعالجة بفيتامين سي، لوحظ تحسن لافت للنظر في تلك التغييرات.

**الاستنتاجات:**

استطاعت المعالجة التالية بفيتامين سي أن تحسن كل التغييرات الناجمة عن تسمم الكبد بكل من الديكلوفيناك صوديوم والنابروكسين.

## Introduction

Nonsteroidal anti-inflammatory drugs are the most popular medications worldwide in view of their diverse uses as antipyretics, analgesics, anti-cancer and anti-inflammatory drugs, as well as lately as a potential anti-severity novel coronavirus (COVID-19) drug.^1^^,^^2^ Nonsteroidal anti-inflammatory drugs, including ibuprofen and diclofenac, may prevent COVID-19 complications and enhance recovery within a short time when they are used early in COVID-19 treatment.^3^ In addition, naproxen may be a perfect candidate to manage COVID-19 in the future.^4^ Despite these clinical benefits, the risks of frequent and long-term use of these drugs should not be disregarded. Hepatotoxicity is among their numerous risks; nonsteroidal drug-induced hepatotoxicity is mostly related to diclofenac.^1^^,^^5^ On the other hand, naproxen-induced hepatotoxicity has rarely been documented, and its toxicity is mediated by oxidative stress.^6^^,^^7^ Similarly, oxidative stress is also linked to diclofenac-induced hepatotoxicity, and Jung et al. recently demonstrated the underlying mechanism.^8^ These researchers found that diclofenac promotes oxidative stress production and impairs lysosome function, thereby suppressing autophagy and mitochondrial dysfunction, which is responsible for hepatotoxicity. Hepatocytes’ mitochondria are the main targets of oxidative stress induced by diclofenac and its metabolites, leading to the inhibition of adenosine triphosphate (ATP) production and alteration in mitochondrial function.^9^^,^^10^ Hepatocyte damage caused by drug toxicity can impact the lipid profile because the liver plays an important role in lipid metabolism and lipoprotein biosynthesis. Noticeably, abnormal lipid profiles have been reported to correlate with the progression of liver damage caused by cirrhosis. In addition, altered lipid profiles are associated with severe hepatitis, chronic liver diseases, and hepatic failure due to impairment of lipoprotein biosynthesis.^11^

Free radical molecules have unpaired electrons; therefore, they can react with other molecules. These free radicals are vital molecular components called reactive oxygen species, which have essential roles in cell signalling, gene expression, apoptosis, and ion transportation. However, high levels of reactive oxygen species cause oxidative stress, resulting in significant damage to many molecules, including DNA, RNA, proteins, and lipids. Antioxidant molecules protect against cellular damage due to the harmful effects of oxidative stress in the body. Vitamin C or ascorbic acid is a water-soluble vitamin that plays an essential role in various functions in the body and has been investigated as an effective antioxidant. Vitamin C has potent reducing properties due to its hydroxyl groups in the lactone ring that act as proton and electron donors. It can decrease oxidative damage either **i)** directly by reacting with or neutralizing hydroxyl, alkoxyl, and lipid peroxyl radicals or **ii)** indirectly by reducing the activity of enzymes that catalyse free radical-generating reactions or increasing the activity of intracellular antioxidant enzymes.^12^^,^^13^ It is worth noting that mega doses of vitamin C could improve the histopathological features of non-alcoholic steatohepatitis.^14^ In addition, vitamin C is capable of significantly minimising the hepatotoxic effects of both mercury and cadmium in rabbits' livers.^15^ A study has suggested vitamin C's potential protective role regarding oxidative stress promoted by diclofenac sodium, which has been found to cause acute nephrotoxicity in rats' kidneys.^16^ Furthermore, vitamin C is the most commonly consumed daily dietary supplement.^13^ The above facts encouraged us to propose this study to investigate the possibility of post-treatment with vitamin C in the amelioration of the deleterious effects of diclofenac sodium and naproxen in the livers of prepubertal rats and highlight these effects' relationship with the lipid profile.

## Materials and Methods

### The animals and study design

A total of 40 premature female albino rats aged 5 weeks old with a weight in the range of 60 g–70 g were housed under appropriate conditions at Pharmacy College, Basrah University.

The current study was conducted in two parts:

**Part I:** To assess the hepatotoxic effects of nonsteroidal drugs, the animals were divided into three groups, as follows: 10 rats in the control group, 15 rats in the diclofenac sodium group, and 15 rats in the naproxen group. They received daily oral drug administration by gavage with 0.1 ml of 5% dimethyl sulfoxide, 5 mg per kg (body weight) and 50 mg per kg, respectively. The duration of the drug administration was 21 days. Then five rats from each drug-adminstered group were anaesthetised and dissected and the results were compared with those obtained for five rats from the control group after the end of the administration period.

**Part II:** To assess the ameliorative role of post-treatment with vitamin C, the remaining animals in both drug-administered groups (five rats per group) were orally (by gavage) post-treated daily with 25 mg/kg of vitamin C (according to Djurasevic et al.^19^) for 21 days. The other five rats in each drug-administered group were not post-treated with vitamin C and were considered as positive controls in addition to the five rats in the control group as a negative control. At the end of the 3 weeks, all animals (five rats in each group) were anaesthetised and dissected.

Thus, the total duration of this study was 42 days (21 days for nonsteroidal drug administration and 21 days for post-treatment with vitamin C. Notably, the dose of diclofenac sodium was approximately one-tenth that of oral LD50 in rats (5 mg/kg), which was previously used as the therapeutic dose for the treatment of inflammation.^17^ The naproxen dose (50 mg/kg) was also one-tenth that of oral LD50, the conventional anti-inflammatory dose in rats.^18^ To prepare the doses, the two drugs were dissolved in dimethyl sulfoxide.

### Biochemical evaluation

To obtain serum, blood samples were collected by cardiac puncture. The blood tubes without anticoagulant were centrifuged at 4000 rpm for 20 min using a cold centrifuge (−4 °C) (Genex, Florida, USA). Analytic commercial kits (JOURILABS, Ethiopia) were used, and the manufacturer's instructions were precisely followed to evaluate the levels of aspartate and alanine aminotransferases, triglycerides, high-density lipoprotein, and total cholesterol; the Friedwald formula Gebrie et al.^20^ mentioned was applied to calculate the levels of low- and very low-density lipoproteins.

### Histopathological investigation

After dissection, each rat's liver was removed and preserved in 10% buffered formaldehyde, and the liver specimens were dehydrated and cleared. The specimens were immersed in paraffin to obtain wax blocks that were ready to cut using a microtome. The liver sections (5 μm in thickness) were fixed on slides and stained with haematoxylin and eosin for histological examination using a light microscope equipped with a digital camera (LCD light microscope).^21^

### Statistical analysis

The biochemical data were analysed using GraphPad Prism software (version 5.0, California, USA) and tested via analysis of variance (ANOVA), Bonferroni's post-test, and unpaired t-test. Significance was set at a *p*-value < 0.05.

## Results

### The liver enzymes

**Figure 1:**
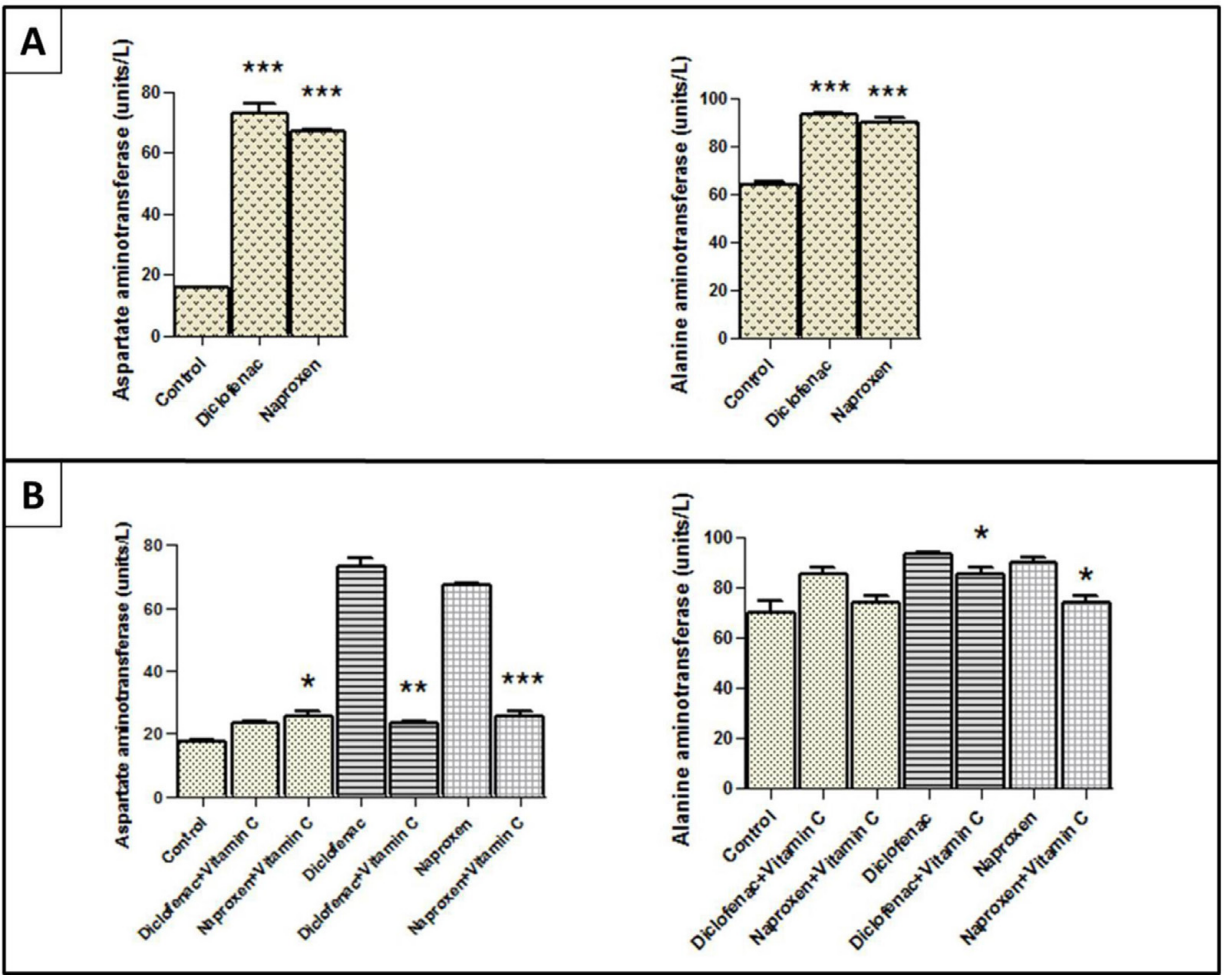
**The levels of liver enzymes in the serum of rats administered nonsteroidal anti-inflammatory drugs before and after post-treatment with vitamin C compared with those in control rats.** (A) The levels of aspartate aminotransferase and alanine aminotransferase in the diclofenac sodium and naproxen groups before post-treatment with vitamin C. (B) Aspartate and alanine aminotransferase levels in the diclofenac sodium and naproxen groups after post-treatment with vitamin C. The stars express the significance; ∗ indicates *p* < 0.05, ∗∗ indicate *p* < 0.01, and ∗∗∗ indicate *p* < 0.001; msean ± SEM was considered.

The serum levels of aspartate and alanine aminotransferases were significantly elevated (*p* < 0.001) in rats that were administered diclofenac sodium and naproxen compared with those control rats (Figure 1A). However, Figure 1B shows that the levels of these two enzymes were slightly elevated in the same diclofenac and naproxen groups after post-treatment with vitamin C. Notably, the enzyme levels decreased significantly after post-treatment with vitamin C compared with those before post-treatment (Figure 1B).

### Lipid profile

**Figure 2:**
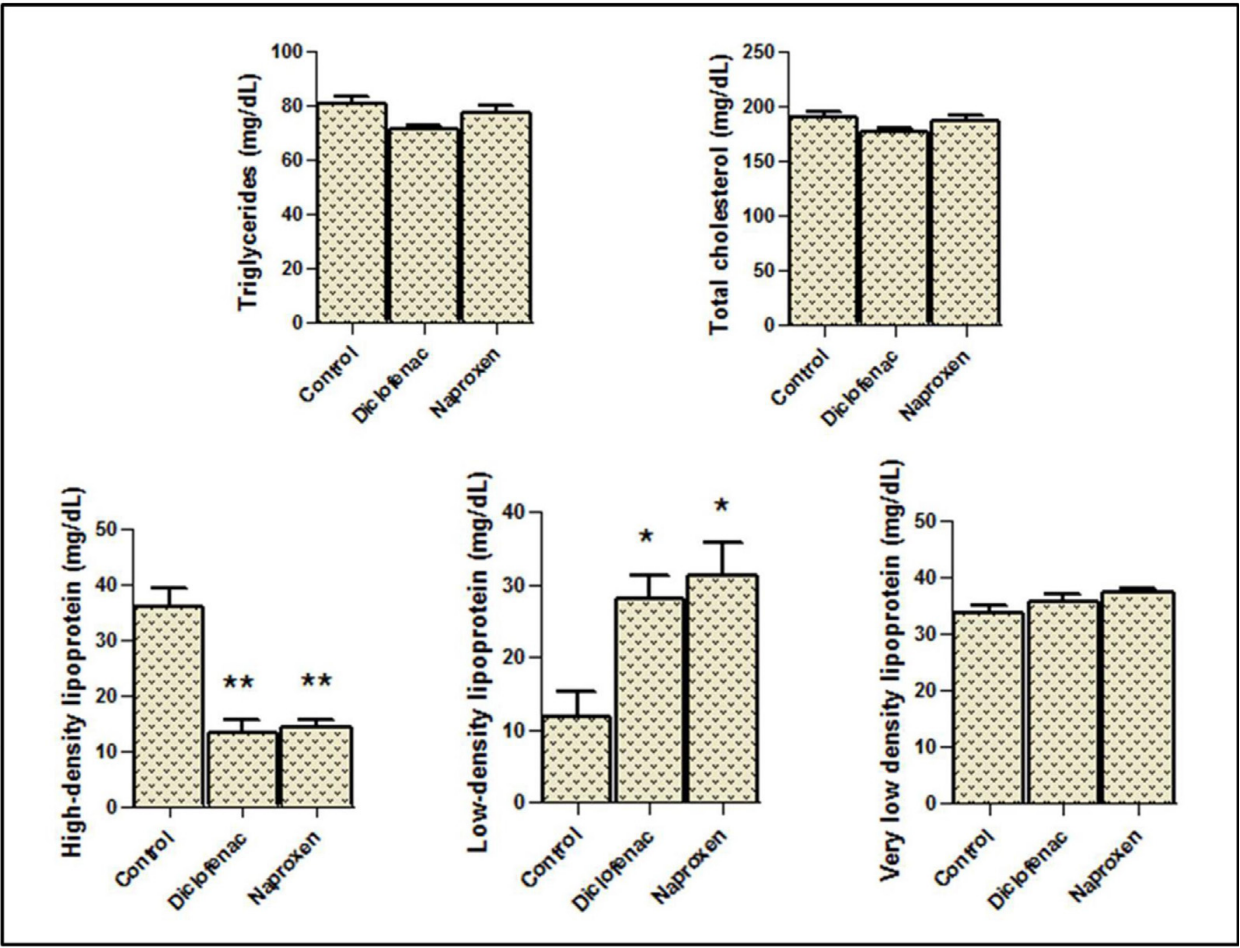
**Serum levels of the lipids in the rats administered diclofenac sodium and naproxen.** The stars express the significance; ∗ indicates *p* < 0.05, ∗∗ indicate *p* < 0.01; mean ± SEM was considered.

In general, statistical analysis of the lipid profile data showed nonsignificant effects of diclofenac and naproxen on the levels of total cholesterol, very low-density lipoprotein, and triglycerides in the serum, but not for high- and low-density lipoproteins, which were significantly affected, as shown in Figure (2). High-density lipoprotein levels decreased significantly (*p* < 0.01) in the diclofenac and naproxen groups compared with those in the control group. By contrast, low-density lipoprotein levels increased significantly (*p* < 0.05) in the drug groups compared with those in the control group (Figure 2).

**Figure 3:**
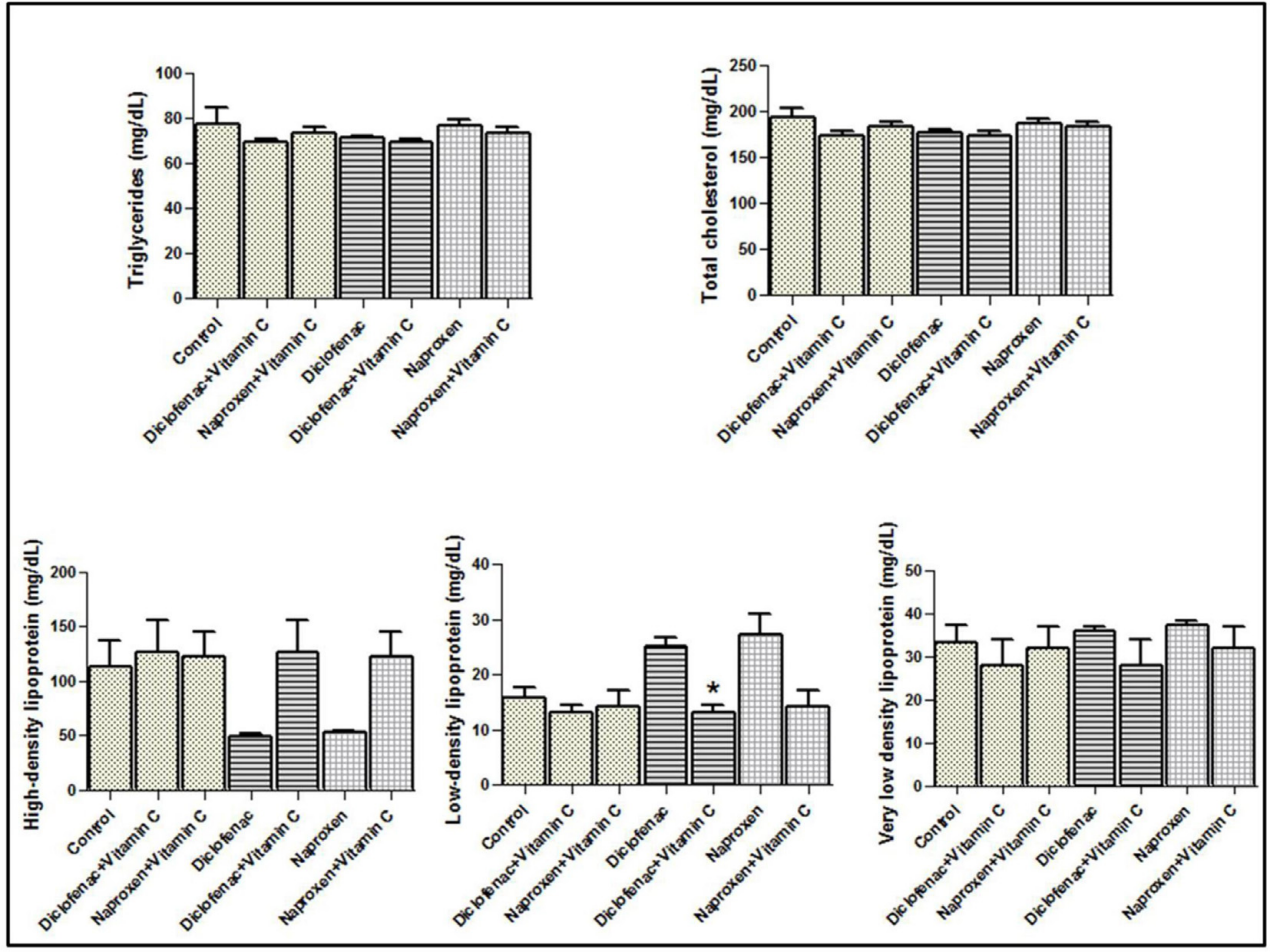
**Serum levels of the lipids in the vitamin C-treated rats after diclofenac sodium and naproxen administration.** The star indicates the significance at *p* < 0.05; mean ± SEM was considered.

When the rats that were administered nonsteroidal drugs were post-treated with vitamin C, the serum levels of the lipid parameters mentioned above did not differ significantly from those in the control group (Figure 3). However, compared with the groups that did not received vitamin C post-treatment, the two groups that receive vitamin C post-treatment showed noticeable amelioration in the levels of both high- and low-density lipoproteins (Figure 3).

### Liver histopathology

**Figure 4:**
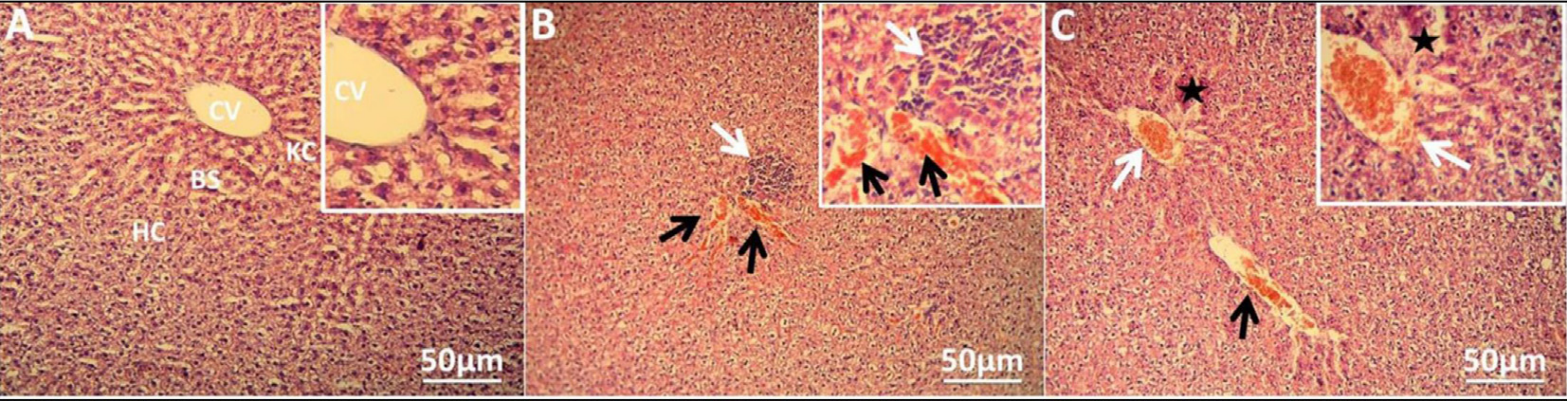
**Hepatic injuries in the rats' livers induced by nonsteroidal anti-inflammatory drug administration.** (A) Photomicrograph of control group depicting intact histological structure with normal central vein, normal sinusoids, and surrounding hepatocytes in the parenchyma. (B) Photomicrograph of rats administered diclofenac sodium displaying congestion in sinusoids (black arrow) with inflammatory cell infiltration in the portal area (white arrow). (C) Photomicrograph of rats administered naproxen showing degeneration of the hepatocytes (star) in the hepatic parenchyma associated with congestion in the central vein (white arrow) and sinusoids (black arrow). Stain: haematoxylin and eosin; magnifications:×100 and ×400 for main and inset images respectively. (CV: central vein, HC: hepatocytes, KC: Kupffer cells, BS: blood sinusoids).

Histological examination of the control liver revealed intact liver tissue, distinguished by well-organised hepatic lobules with hepatic strands of radially regular hepatocytes enclosed in the central vein (Figure 4A). In rats that were administered nonsteroidal anti-inflammatory drugs, several pathological alterations were observed, including dilation and congestion in the blood sinusoids and central vein, and infiltration of the inflammatory cells in the portal area, which were observed in the liver sections of diclofenac-administered rats (Figure 4B). In addition, the naproxen group showed degeneration in the hepatocytes of the liver parenchyma associated with congestion in both the central vein and sinusoids (Figure 4C).

**Figure 5:**
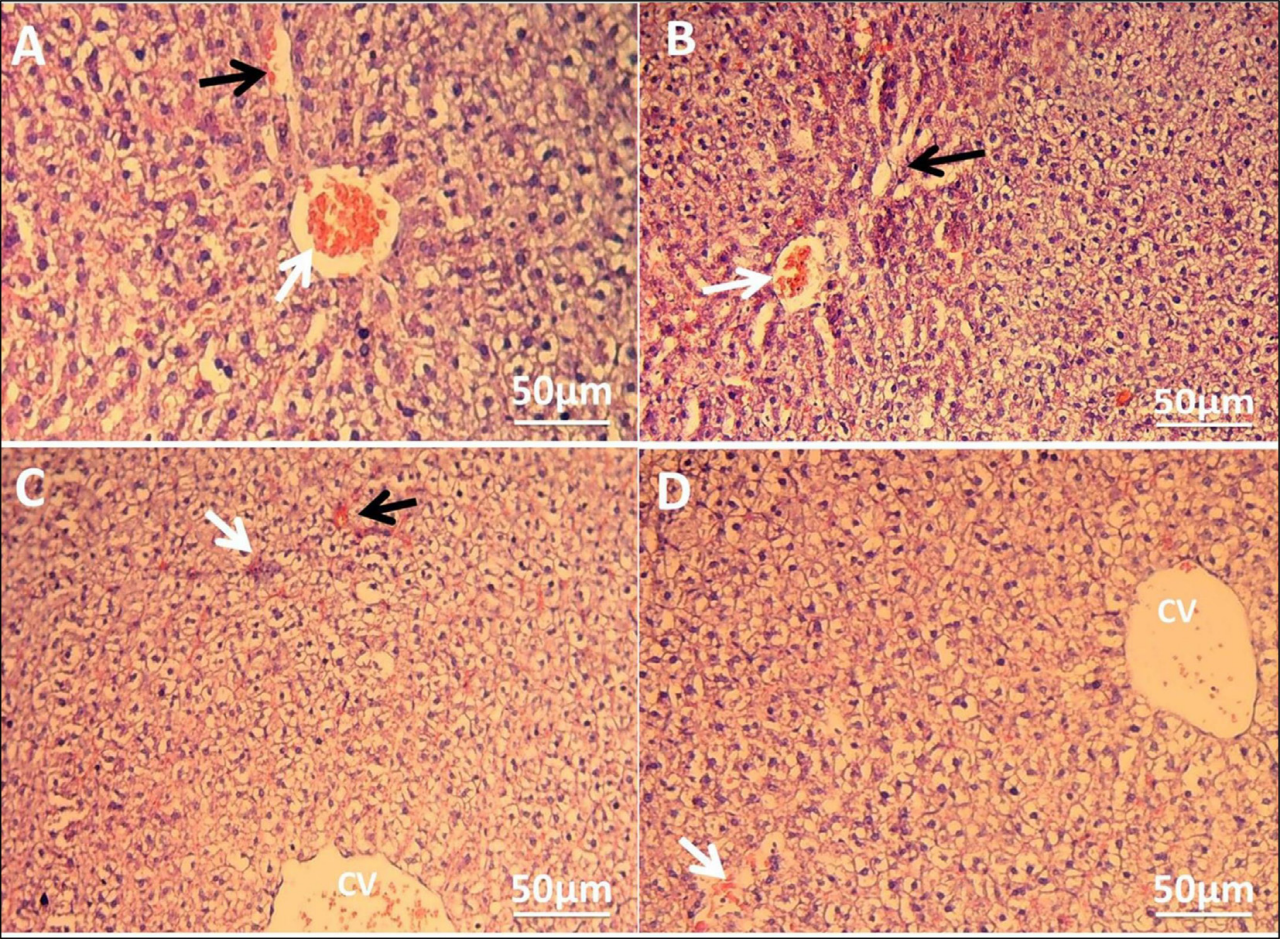
**Ameliorative effects of vitamin C on damage caused by nonsteroidal anti-inflammatory drugs in liver histology.** (A) Photomicrograph of diclofenac-administered rats without vitamin C post-treatment displaying central vein congestion (white arrow) and congestion and dilation in blood sinusoids (black arrow). (B) Photomicrograph of rats administered naproxen without vitamin C post-treatment showing distortion of normal hepatic arrangement, congestion in the central vein (white arrow), and degeneration in the hepatocytes (black arrow). (C) Photomicrograph of diclofenac group with vitamin C post-treatment showing slight central vein congestion (CV) and a reduction of inflammatory cell infiltration (white arrow). In addition, mild congestion was observed in the sinusoids (black arrow). (D) Photomicrograph of naproxen group with vitamin C post-treatment displaying normal radial arrangement of hepatocytes around the central vein with mild congestion and dilatation of hepatic sinusoids (white arrow). Haematoxylin and eosin stain, magnification of images; ×100.

After the conclusion of the period of vitamin C post-treatment, the rats in the diclofenac and naproxen groups without vitamin C (positive controls) still had different histopathological signs of hepatotoxicity, such as a congested central vein, dilated and congested hepatic sinusoids, inflammatory cell infiltration, and disruption of normal hepatic construction with hepatocyte degeneration (Figure 5A and B). Interestingly, the liver tissue of vitamin C post-treated rats, compared to those without vitamin C post-treatment, microscopically showed slight congestion in the central vein and sinusoids, as well as a remarkable reduction in inflammatory cell infiltration in the diclofenac group post-treated with vitamin C (Figure 5C). In addition, the naproxen group post-treated with vitamin C exhibited a normal radial arrangement of hepatocytes around the central vein and mild hepatic damage represented by slight congestion and dilatation of hepatic sinusoids and slight degeneration changes (Figure 5D).

## Discussion

Drugs can cause hepatic injury; the most common drugs related to hepatotoxicity are nonsteroidal anti-inflammatory drugs.^22^ The clinical features of liver damage induced by nonsteroidal anti-inflammatory drugs extend from an asymptomatic state, an impermanent state, and hypertransaminasemia to liver failure.^23^ Hepatic enzymes commonly related to hepatotoxicity are aspartate transaminase and alanine transaminase, which serve as biomarkers for hepatic toxicity.^1^ The serum levels of these transaminases or aminotransferases were significantly elevated in the present study, indicating that diclofenac sodium and naproxen induced hepatotoxicity. Uysal et al. reported significantly high serum transaminases as indicators of hepatic damage induced by paracetamol.^24^ Diclofenac sodium-induced hepatotoxicity significantly elevates serum aspartate and alanine aminotransferase activities in rats.^25^, ^26^, ^27^, ^28^ Moreover, diclofenac metabolites can significantly and rapidly increase serum levels of alanine transaminase, an indicator of acute liver injury, in mice.^29^ Regarding naproxen, a study^7^ reported significant elevation in the levels of aspartate and alanine transaminases induced by naproxen in rats. Another study^30^ reported a significant increase in the levels of these liver enzymes in patients receiving 250 mg/day of naproxen. Our study demonstrated that vitamin C post-treatment can reduce the elevation in liver aminotransferase levels caused by diclofenac sodium and naproxen. This result was in agreement with the findings of Alhusaini et al.^31^ who reported that vitamin C treatment significantly attenuated the levels of hepatotoxicity biomarkers (aspartate and alanine aminotransferases), which were elevated by lead acetate in rats. In addition, vitamin C exerted an ameliorative effect on emamectin benzoate-induced hepatotoxicity in rats, as it reduces biochemical and histopathological abnormalities.^32^ Moreover, treatment with 250 mg/kg of vitamin C for 7 days could protect against carbon tetrachloride-induced hepatotoxicity in rats.^33^

Regarding the effects of diclofenac sodium and naproxen on the lipid profile, the current study showed no significant effects of diclofenac sodium and naproxen on lipid parameters, except for high- and low-density lipoproteins (good and bad cholesterols, respectively). The effect was clear and significant for both high- and low-density lipoproteins. High-dose diclofenac administration (10 mg/kg) can induce hepatic injury in rats and disrupt the lipid profile.^34^ Nouri et al.^35^ reported similar results regarding a significant reduction in high-density lipoproteins and significant augmentation in low-density lipoproteins associated with hepatotoxicity induced by diclofenac in rats. However, the significant reduction in triglycerides and total cholesterol disagrees with our results, which show nonsignificant reduction in these parameters. The significant decrease in high-density lipoprotein levels and the significant increase in low-density lipoprotein levels are also caused by d-galactosamine-induced hepatotoxicity in rats.^36^ It is worth noting that there are no available studies on the lipid profile related to liver injury induced by naproxen to support the interpretation of the results. A previous study^35^ reported a significant decrease in the vitamin C content in the liver caused by diclofenac administration-induced hepatotoxicity. This decreased level of vitamin C is associated with a changed lipid profile.^35^ Perhaps there is a correlation between the two that may explain the ameliorative effects of vitamin C on both high- and low-density lipoprotein levels after post-treatment with vitamin C in the current study. Alternatively, the altered lipid profile may be related to oxidative stress-mediated hepatotoxicity, and vitamin C acts as an antioxidant to reduce oxidative stress and subsequently ameliorate the lipid profile. Indeed, Hillstrom et al.^37^ demonstrated that vitamin C can control lipid oxidation in high-density lipoproteins, which, in turn, prevents oxidative alteration in low-density lipoproteins. Thus, vitamin C could modulate the levels of both high- and low-density lipoproteins, which were significantly altered by oxidative stress-mediated hepatotoxicity because these lipoproteins are susceptible to oxidation.^37^

Wang et al.^38^ studied the potential mechanism underlying the beneficial effects of vitamin C on the lipid profile and proved that vitamin C can regulate serum levels of high- and low-density lipoproteins through activation of the expression of the low-density lipoprotein receptor in the liver by inhibiting expression of the proprotein convertase subtilisin/kexin 9. Thus, vitamin C supplementation is suggested to ameliorate lipid profiles in vitamin C-deficient species.

In the context of the histopathological effects of diclofenac sodium and naproxen on rat livers, the hepatic micrographs in the current investigation illustrated hepatotoxic effects induced by both diclofenac sodium and naproxen. Diclofenac sodium administration led to several injuries, including dilation and congestion in the blood sinusoids and central vein, and inflammatory cell infiltration in the portal area. Previous studies^26^^,^^28^^,^^35^ also photographed inflammatory cell infiltration induced by diclofenac in rats' liver tissue. This infiltration is leucocyte migration, which indicates an inflammatory response stimulated by tissue damage.^28^ Our observations of congestion in the blood sinusoids and central vein were in agreement with those Aljuhani et al.^21^ observed. On other hand, no findings concerning the histopathological effects of naproxen in the liver can be included in this discussion because naproxen-induced hepatotoxicity has rarely been studied. In this study, liver damage represented by hepatocyte degeneration associated with congestion in both the central vein and sinusoids in rats administered naproxen could refer to hepatotoxicity. The hepatotoxicity induced by diclofenac sodium and naproxen could be mediated by oxidative stress. Some studies have mentioned that administration of both drugs induces oxidative stress that causes liver toxicity.^7^^,^^26^^,^^28^ Therefore, post-treatment with vitamin C as an antioxidant led to amelioration of the pathological manifestations induced by both diclofenac sodium and naproxen. Vitamin C can ameliorate liver tissue damage induced by dexmedetomidine because it protects against toxic free radicals and minimises lipid peroxidation-mediated cellular damage.^39^ Vitamin C can also improve the hepatotoxic effects of fenitrothion in rats by reducing oxidative stress.^40^ Furthermore, vitamin C can suppress the inflammatory response and endoplasmic reticulum stress-mediated liver damage induced by perfluorooctane sulfonate in mice.^41^ In addition, Khaldoun Oularbi et al.^32^ demonstrated the ameliorative effect of vitamin C on signs of hepatic architectural damage represented by infiltration of inflammatory cells, steatosis of hepatocytes, and necrosis induced by emamectin benzoate in rats. The benefits of vitamin C's biological mechanism in combating liver injury are the regulation of inflammatory reactions and apoptosis.^42^

## Conclusions

In conclusion, the biochemical and histopathological analyses performed in the present study demonstrated the hepatotoxic effects induced by nonsteroidal drugs in prepubertal rats. The lipid profile was significantly altered in association with hepatotoxicity. Interestingly, post-treatment with vitamin C following nonsteroidal administration could be beneficial for improvement of the alterations in serum liver biomarkers, hepatic tissue, and the lipid profile.

## Recommendation

In view of our findings, it can be predicted that vitamin C may reduce also the cardiovascular risks resulted from the toxicity of nonsteroidal drugs and strongly associated with high serum levels of low density lipoproteins through reduction of these lipids by vitamin C. This prediction serves as a proposal for future studies.

## Source of funding

This research did not receive any specific grant from funding agencies in the public, commercial, or not-for-profit sectors.

## Conflict of interest

The authors have no conflict of interest to declare.

## Ethical approval

Approval for animal use was obtained from the Iraqi Ethical Committee of the Animal Research of Basrah University (Approval No. 3108002 in 05-04-2021), and the guidelines of the National Research Council for the laboratory animals Care and Use of Laboratory Animals were precisely followed for providing the animals with a good quality of life including food, water, housing, acclimatisation, cleaning, freedom of movement, and safety from predation and injury. During and after using the animals in the experiments of this study, unnecessary pain (refinement), distress, and discomfort were avoided.

## Authors contributions

THWA designed the study, and MNA conducted the methodology. MNA, THWA, and RSA provided the materials. THWA and RSA performed analysed and interpreted the results. THWA drafted the manuscript. All authors have critically reviewed and approved the final draft and are responsible for the content and similarity index of the manuscript.
